# Liver transplantation in a child with sclerosing cholangitis due to Langerhans cell histiocytosis: a case report

**DOI:** 10.3389/fped.2024.1414104

**Published:** 2024-11-07

**Authors:** Xue-Lian Wang, Chun-Xiao Fang, Min-Xia Chen, Hua-Mei Yang, Lan-Hui She, Yu Gong, Yi Xu, Wei-Qiang Xiao, Jin-Sheng Tian, Bin Ai, Li Huang, Xu-Fang Li

**Affiliations:** ^1^ Department of Infectious Diseases, Guangzhou Women and Children’s Medical Center, Guangzhou Medical University, Guangzhou, China; ^2^ Department of Radiology, Guangzhou Women and Children’s Medical Center, Guangzhou Medical University, Guangzhou, China

**Keywords:** Langerhans cell histiocytosis, sclerosing cholangitis, cholestasis, liver cirrhosis, liver transplantation

## Abstract

**Background:**

Langerhans cell histiocytosis (LCH) is a systemic neoplasia with diverse clinical manifestations, predominantly affecting bone and skin. However, in children, LCH presenting primarily with cholestasis is rare.

**Case summary:**

We present the case of a 22-month-old boy who was admitted to our hospital with a history of intermittent fever and abdominal distension for over 2 months, and jaundice for over 1 month. Prior to admission, the child had been managed with anti-infective and anti-inflammatory drugs and supportive care at multiple hospitals without significant improvement. He was then referred to our facility for further treatment. Upon admission, a series of laboratory tests, imaging studies, and pathological examinations were conducted, revealing the presence of diabetes insipidus, sclerosing cholangitis (SC), and liver cirrhosis. These findings led to a clinical diagnosis of LCH. Given the absence of definitive pathological evidence, his progression to decompensated liver cirrhosis and his pronounced growth retardation, the child was deemed a candidate for living donor liver transplantation. Following the liver transplant, pathological examination of the explanted liver tissue confirmed the clinical diagnosis of LCH. The child received postoperative chemotherapy, which resolved his systemic symptoms and normalized liver function. There was no evidence of LCH recurrence. The symptoms of diabetes insipidus were controlled with desmopressin acetate, however, the existing destructive lesions in the pituitary gland persisted.

**Conclusion:**

Sclerosing cholangitis is a late and severe form of liver involvement in LCH that may be accompanied by lesions in other tissues or organs. Along with pathological evidence, a diagnosis should be made based on characteristic rashes, osteolytic lesions, and thickening of the pituitary stalk. In children with LCH complicated by SC for whom pathological diagnostic evidence cannot be obtained, liver transplantation may be considered once signs of decompensated liver cirrhosis appear, followed by systemic chemotherapy to control active disease. This strategy represents a therapeutic approach with the potential to achieve a better prognosis in children with LCH.

## Introduction

Langerhans cell histiocytosis (LCH) is a rare neoplasia most commonly characterized by osteolytic lesions, rashes, and diabetes insipidus. A definitive diagnosis is made by positive immunohistochemical staining for CD1a, CD207, and S100 (1). Liver involvement in LCH can range from mild transaminitis to end-stage liver disease (ESLD). Sclerosing cholangitis (SC) is a late and severe manifestation of liver involvement, which can progress to biliary cirrhosis and liver failure, ultimately necessitating liver transplantation (2). However, there is little existing research on the role of liver transplantation in the treatment of LCH complicated by SC. Herein, we report the case of a child with LCH with cholestasis as the first manifestation, who underwent liver transplantation due to the development of decompensated cirrhosis.

## Case presentation

A 22-month-old boy was admitted to Guangzhou Women and Children's Medical Center with intermittent fever accompanied by abdominal distention for over 2 months and jaundice for over 1 month. Prior to admission, the child had been found to have elevated leukocytes, as well as abnormally high liver and bile enzymes (aspartate aminotransferase 95.7 U/L, alanine aminotransferase 65.1 U/L, γ-glutamyl transferase 455 U/L, total bilirubin 54 umol/L, direct bilirubin 53.9 umol/L, albumin 28.3 g/L, total bile acids 177 umol/L). Computed tomography (CT) and magnetic resonance imaging (MRI) imaging revealed multiple intrahepatic bile duct dilatations, cholangitis, cholecystitis, hepatosplenomegaly, and a small amount of ascites. Medical exome testing showed no abnormalities. Despite being managed for over a month with anti-infective and anti-inflammatory drugs and supportive care, the child continued to have recurrent fever, persistent jaundice and progressive worsening of abdominal distention. The child had mild jaundice in the neonatal period, which resolved spontaneously at 1 month of age. The child's motor development was delayed compared to peers (e.g., lifting head at 4 months, sitting upright at 9 months, crawling at 10 months, standing at 18 months, and currently standing with support and an unstable gait); there was no family history of related disease.

Upon admission, a comprehensive physical examination was performed. He was 80 cm (P 3.7) in height and weighed 11.2 kg (P 39.4). There was mild jaundice of the skin and sclerae, with no rash. No palpable enlargement of the superficial lymph nodes was noted. The abdomen was distended, soft, with a navel circumference of 51 cm and maximum abdominal circumference of 53 cm. The liver was palpable 12 cm below the right midclavicular line, and the spleen was palpable 6 cm below the left midclavicular line, both of which were tough upon palpation. No other abnormalities were documented during physical examination.

Laboratory tests confirmed the presence of elevated liver and bile enzymes (alanine aminotransferase 114 U/L, aspartate aminotransferase 155 U/L, total bilirubin 47.2 umol/L, direct bilirubin 34.8 umol/L, albumin 30 g/L, alkaline phosphatase 1,262 U/L, γ-glutamyl transferase 437 U/L, total bile acids 122 umol/L). A differential blood count revealed leukocytosis (17.9 × 10^9 ^/L), 58% neutrophil content, and microcytic hypochromic anemia. An immunoglobulin (Ig) panel revealed elevated IgE (165.00 IU/ml), with no other abnormalities. Coagulation function (prothrombin time 12.5 s, international normalized ratio 0.95) and bone marrow cytology were normal. Tests for hepatotropic pathogens (hepatitis viruses, cytomegalovirus, Epstein-Barr virus, syphilis, human immunodeficiency virus, human parvovirus B19, herpes simplex virus, rubella virus, adenoviruses, and enteroviruses) were all negative. Tests for autoimmune and heritable metabolic diseases were likewise negative.

**Figure 1:**
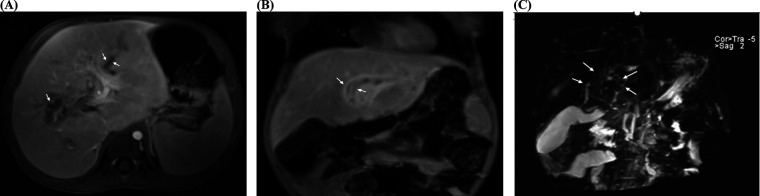
Magnetic resonance imaging (MRI) and magnetic resonance cholangiopancreatography (MRCP) of a 22-month old patient with LCH-associated sclerosing cholangitis (SC). **(A)** The dilated intrahepatic bile duct (white arrows) with bile retention and obstruction (transverse T1WI). **(B)** The dilated intrahepatic bile duct (white arrows) with diffuse wall thickening (coronal T1WI). **(C)** Left and right hepatic ducts; common bile duct not shown (the area indicated by the white arrow does not exhibit the “Y” shaped configuration). T1WI, T1-weighted image.

Hepatic MRI and magnetic resonance cholangiopancreatography (MRCP) revealed diffuse enlargement of the liver with abnormal signals suggestive of cholestatic hepatitis. Specifically, there was diffuse thickening of the walls of the intrahepatic bile ducts, segmental dilatation of the intrahepatic bile ducts, bile retention and obstruction within the bile ducts suggesting sclerosing cholangitis (Figure 1). There was evidence of splenomegaly with minimal abdominal ascites. Liver elastography revealed a stiffness of 36 kPa, indicating severe liver cirrhosis. Abdominal vascular ultrasound revealed a normal appearance of the abdominal aorta, inferior vena cava, superior mesenteric artery, main portal vein, and splenic vein, with unobstructed blood flow and no significant abnormalities. The child's family repeatedly refused to perform a liver biopsy during the hospital stay.

**Figure 2:**
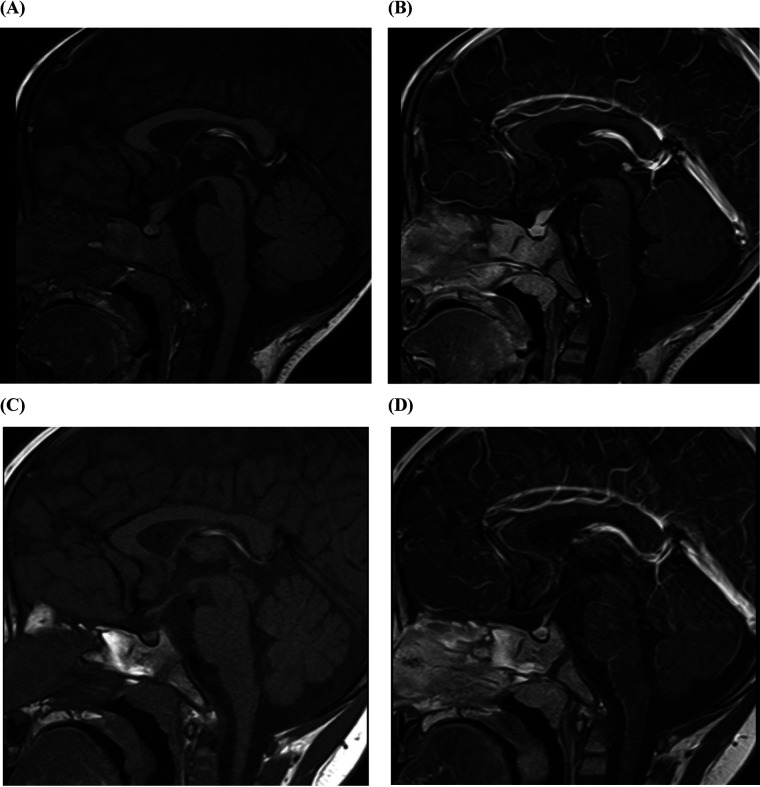
Pituitary MRI of a 22-month old patient with LCH presenting with SC and diabetes insipidus. **(A)** The pituitary stalk is thickened, with a width of approximately 4.1 mm and an anterior-posterior diameter of 4.4 mm. On T1WI plain scan, the high signal of the posterior pituitary lobe disappears. **(B)** Enhanced T2WI pituitary enhancement is uniform and consistent, with no abnormal foci of enhancement. **(C)** At 21-mo follow-up, the pituitary stalk had a width of about 2.5 mm and an anterior posterior diameter of 3.0 mm. On T1WI plain scan, the high signal of the posterior pituitary lobe disappears as before. **(D)** At 21-mo follow-up, enhanced T2WI pituitary enhancement is uniform and consistent, with no abnormal foci of enhancement. T1WI, T1-weighted image; T2WI, T2-weighted image.

During the MRCP dehydration process, a particular phenomenon drew special attention: the child was crying, expressing a desire to drink water, and comforted when offered water to drink. Upon revisiting the medical history, his mother recalled that over the past 3 months, the child had been consuming a significant amount of water daily and had experienced nocturia. Consequently, we recorded his 24 h oral fluid intake and urine output over the next 3 days. Polydipsia and polyuria were confirmed, with his fluid intake reaching 6–10 L/(m^2^·d) and urine output reaching 7–11 L/(m^2^·d). Plasma osmolality fluctuated between 270 and 277 mOsm/kg, and urinalysis revealed a specific gravity of 1.003. Blood glucose, blood gas and electrolyte analysis, and renal function were found to be within normal limits. The parents did not consent to a water deprivation/vasopressin test. Therefore, we recommended a complete MRI of the head and pituitary gland, which demonstrated the disappearance of the high signal in the neurohypophysis and thickening of the pituitary stalk (Figures 2A,B). In conjunction with the child's symptoms of polydipsia and polyuria, central diabetes insipidus was considered.

Given the patient's coexisting sclerosing cholangitis, liver cirrhosis, and diabetes insipidus, the physician strongly suspected LCH. However, the absence of characteristic rashes, osteolytic lesions on whole-body radiographs, and the inability to obtain a liver biopsy prevented the pathological diagnosis of LCH and complicated the child's subsequent treatment. Despite his less-urgent Child-Pugh classification (B) and Pediatric End-Stage Liver Disease (PELD) score (3), the child's sclerosing cholangitis, decompensated liver cirrhosis, and significant growth retardation qualified him for a medically urgent liver transplant. Consequently, we referred him to the Liver Transplantation Center at Shanghai Children's Hospital for surgical treatment.

In a subsequent follow-up, we learned that the child underwent two liver biopsies, both of which revealed signs of cholestasis and cirrhosis with no characteristic changes of LCH noted on immunohistochemical staining. The child successfully underwent a living-donor liver transplant from his mother 1 week post-biopsy. During the surgery, the child's entire liver and extrahepatic bile ducts were resected, and cholangiojejunostomy Roux-en-Y was performed. Regrettably, pathological images were not obtained.

The excised liver was enlarged, grayish-yellow to grayish-green on the cut surface, and had an intermediate texture. Focal infiltration of Langerhans cells was observed on histological examination. Moreover, immunohistochemical staining indicated that the infiltrating cells were positive for CD1a, S-100, and Langerin, but negative for BRAF V600E, CD21, CD23, CD34, CD 68/PG-M1, myeloperoxidase (MPO), and terminal deoxynucleotidyl transferase (TDT), confirming the presence of neoplastic Langerhans cells.

**Figure 3:**
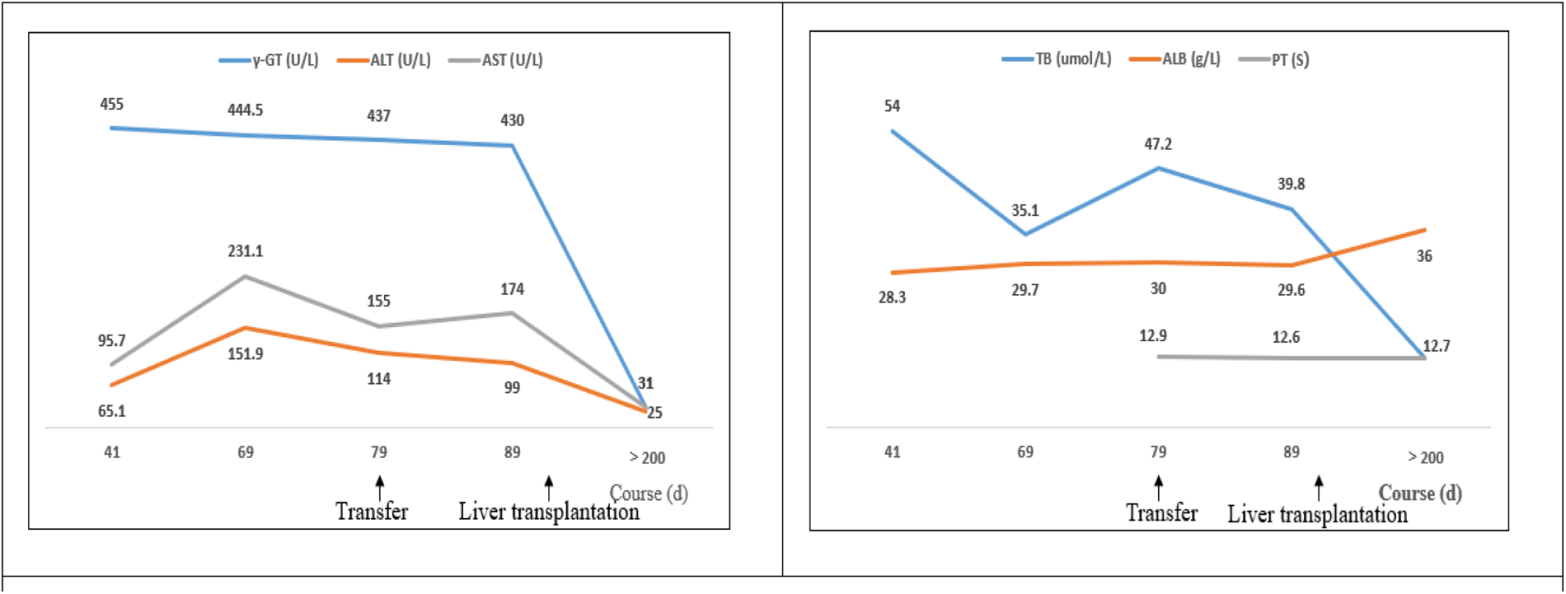
The changes in biochemical parameters of liver function of a 22-month old patient with LCH-associated SC. The patient was found to have abnormally high liver and bile enzymes on day 41 of the documented course of illness; he was referred to our hospital on day 79. A living-donor liver transplantation was performed on day 115. More recent liver function data were not obtained prior to transplantation. ALT, alanine aminotransferase; AST, aspartate aminotransferase; γ-GT, γ-glutamyl transferase; PT, prothrombin time; TB, total bilirubin; ALB, albumin.

The initial chemotherapy protocol included vincristine and prednisone to address systemic symptoms, desmopressin acetate for the treatment of diabetes insipidus, and tacrolimus for the prevention of organ rejection. At a 21-month follow-up, there was a significant improvement in the child's condition, with a return of normal liver function, no evidence of recurrence, and no signs of graft-vs.-host disease (alanine aminotransferase 25 U/L, aspartate aminotransferase 31 U/L, total bilirubin 12.7 umol/L, direct bilirubin 8.1 umol/L, albumin 36 g/L, alkaline phosphatase 231 U/L, γ-glutamyl transferase 43 U/L, total bile acids 7.9 umol/L) (Figure 3). Furthermore, his diabetes insipidus was well-managed, as demonstrated by the improved pituitary imaging (Figures 2C,D).

## Discussion

Langerhans cells originate from the differentiation of monocytes and dendritic cell precursors from bone marrow-derived hematopoietic stem cells and yolk sac cells. The clinical phenotypic heterogeneity of LCH is due to the variability of the formation and tropism of Langerhans cells (1). Approximately 15%–60% of patients with LCH exhibit liver involvement; among these, bile duct involvement is a severe form, manifesting as extrahepatic or intrahepatic sclerosing cholangitis (3). The incidence in children can reach 18%, with a 5-year survival rate of only 25% (4, 5). LCH-associated SC, like other primary and secondary forms of SC, is characterized by the elevation of cholestasis-associated liver enzymes, and the appearance on MRCP of multifocal stenosis and dilation of the intrahepatic and extrahepatic biliary tree (6). A definitive diagnosis requires positive immunohistochemical staining for CD1a, CD207, and S100.

The diagnosis of LCH-associated SC is contingent upon the presence of concurrent extrahepatic infiltration. In a French cohort study, 74% of patients with LCH and liver involvement were diagnosed through biopsies of skin, bone, anorectal tissue, lymph nodes, or lung tissue (7). Characteristic rashes and osteolytic lesions are the most common and most important clinical manifestations for diagnostic purposes (8). The child in this report did not exhibit any characteristic rashes or osteolytic lesions that would suggest LCH. Fortunately, we observed his symptoms of polydipsia and polyuria, and pituitary MRI revealed involvement of the pituitary, leading to a clinical diagnosis of central diabetes insipidus and LCH.

It is noteworthy that liver biopsies from patients with LCH-associated SC may not reveal CD1a^+^ cells but instead show CD1a-negative inflammatory infiltration (7, 9), which may confuse the diagnosis with Rosai Dorfman disease. Attention should therefore be paid to the presence of massive cervical lymphadenopathy, leukocytosis, hypergammaglobulinemia, anemia, and elevated ESR (10). Our patient underwent two liver biopsies, and the resulting immunohistochemistry did not reveal changes characteristic of LCH. This is consistent with existing literature indicating that hepatic biopsy is not an ideal diagnostic for LCH (10, 11). Research suggests that in cases where pathological results are unclear or tissue is unavailable, the possibility of diagnosing LCH can be increased by testing for mutations of *BRAF, MAP2K1* (mitogen-activated protein kinase kinase 1)*, ARAF, CCND1* (cyclin D1), or other components of the mitogen-activated protein kinase (MAPK)/extracellular signal-regulated kinase (ERK) signaling pathway in blood or bone marrow (12–14). However, this approach has not yet been applied clinically and will require further validation.

Standard chemotherapy can effectively reverse the acute hepatic injury caused by early infiltration of Langerhans cells in the liver parenchyma (2). However, for late-stage LCH with hepatic involvement and sclerosis, standard chemotherapy is typically ineffective and may cause severe adverse reactions. For example, vinblastine can exacerbate liver dysfunction and, due to abnormal hepatic drug clearance, may increase drug-related toxicity in extrahepatic sites such as the bone marrow and peripheral nerves (15, 16). In addition, studies have found that most patients with LCH have mutations in the MAPK/ERK signaling pathway. Mutations in *BRAF V600E* and *MAP2K1* are the most common (17), and are related to severe clinical manifestations and a high recurrence rate. In particular, patients with LCH and *BRAF V600E* mutation demonstrate a significant resistance to chemotherapy in their bile duct (18). In clinical trials, MAPK/ERK targeted drugs have benefited patients with chemotherapeutic-resistant LCH (13, 19, 20). In particular, treatment with BRAF inhibitors (e.g., Vemurafenib, Dabrafenib) led to a remission rate of 65%–83%. MEK inhibitors (e.g., Trametinib) have also demonstrated benefits for patients with histiocytic tumors including LCH. However, clinical experience in treating pediatric patients with SC-complicated LCH is currently limited, and future research must investigate the selection of targeted drugs as well as their dosages, regimen, and safety in children.

The prognosis of SC arising from LCH is generally poor. Even with systemic chemotherapy, the damage to the biliary tree may still progress (21). In these children, liver transplantation is the preferred treatment to improve hepatic function (22). However, the optimal timing for liver transplantation in patients with LCH remains uncertain. Premature liver transplantation may carry the risk of disease recurrence in the graft, while delayed transplantation may lead to biliary cirrhosis, portal hypertension, and liver failure (2). There are four categories of clinical management strategies for LCH with hepatic involvement, as follows: LCH in remission with compensated liver disease, LCH in remission with decompensated liver disease, active LCH with compensated liver disease, and active LCH with decompensated liver disease (2). In a recent cohort study conducted in India, it was reported that the overall transplantation and survival rates were 100% in six patients with LCH who underwent liver transplantation according to the management algorithm (23). In the case of our patient, the main reasons for liver transplantation were the irreversible cirrhosis caused by SC, significant growth retardation, the involvement of the pituitary gland, and the absence of a pathological basis for diagnosis. The pathological diagnosis of LCH in the liver specimen post-transplantation confirmed the clinical diagnosis, which bought time to plan and initiate the subsequent chemotherapy. In a similar pediatric case of isolated sclerosing cholangitis caused by LCH that was pathologically confirmed after liver transplantation, the child achieved remission with targeted therapy post-transplant (24). In a different case of LCH limited to the extrahepatic bile ducts that was pathologically diagnosed after liver transplantation, there were no signs of LCH recurrence with postoperative immunosuppressive use (25). These cases illustrate that the treatment of SC-complicated LCH must be individualized to the patient and disease state.

We believe that in children with LCH-associated SC where pathological diagnosis cannot be obtained, once there is evidence of decompensated cirrhosis, and especially with severe growth retardation, portal hypertension, or intractable pruritus, liver transplantation should be considered to restore hepatic function prior to initiating chemotherapy.

## Conclusion

In this report, it was found that children with SC who primarily present with cholestatic symptoms, and for whom imaging studies suggest focal bile duct stenosis and dilation, have a lower likelihood of obtaining a diagnosis of LCH through liver biopsy alone. For children with SC-complicated LCH who cannot obtain a pathological diagnosis and have evidence of decompensated cirrhosis, liver transplantation may be considered before systemic chemotherapy to control active disease post-transplantation. This represents a therapeutic approach that may achieve a better prognosis for pediatric patients with SC-complicated LCH.

## Data Availability

The original contributions presented in the study are included in the article/Supplementary Material, further inquiries can be directed to the corresponding author.
